# Malignant Peripheral Nerve Sheath Tumors in Children with Neurofibromatosis Type 1

**DOI:** 10.1155/2014/843749

**Published:** 2014-09-16

**Authors:** Apostolos Pourtsidis, Dimitrios Doganis, Margarita Baka, Despina Bouhoutsou, Maria Varvoutsi, Maria Synodinou, Panagiota Giamarelou, Helen Kosmidis

**Affiliations:** ^1^Oncology Department, “P. & A. Kyriakou” Children's Hospital, Thivon & Levadias Street, 11527 Athens, Greece; ^2^Radiation Department, “P. & A. Kyriakou” Children's Hospital, Athens, Greece; ^3^Pathology Lab, “P. & A. Kyriakou” Children's Hospital, Athens, Greece

## Abstract

*Purpose*. Malignant peripheral nerve sheath tumors (MPNSTs) are rare in children and account for approximately 5–10% of all soft tissue sarcomas in adults. MPNSTs may occur independently but individuals with neurofibromatosis type 1 (NF1) have a significantly increased risk. Our aim is to present patients with MPNST treated in our department. *Cases and Results*. In this report we present 4 cases of MPNSTs (3 females: 13, 12, and 13 years old and 1 male: 10 years old) arising in patients with NF1. All of them presented with an enlarging mass and pain at diagnosis. Tumor was located in the buttock, the spinal cord, the trunk, and the left leg proximal to the heel. Wide excision of the tumor and radiotherapy were applied to all and adjuvant chemotherapy was given to three of them after the disease was progressed. All four died 32, 18, 10, and 22 months after diagnosis with progressive disease locally and pulmonary metastases in two of them. *Conclusions*. In conclusion, MPNSTs arising in patients with NF1 are high grade sarcomas with short survival. Individuals with NF1 should be followed closely in order to identify early the development of MPNSTs. Aggressive surgery and complete excision significantly improves disease-free survival. The usefulness of radiation therapy in MPNSTs is not determined although all patients will receive radiation therapy at some stage of the disease. The role of chemotherapy is unclear.

## 1. Introduction

Malignant peripheral nerve sheath tumors (MPNSTs) are rare high grade sarcomas with a poor prognosis [1–4]. This group of tumors has been reported as malignant schwannoma, malignant neurilemmoma, neurogenic sarcoma, and neurofibrosarcoma whereas the term “malignant peripheral nerve sheath tumour” is currently preferable [2, 4]. MPNSTs may occur independently or in association with neurofibromatosis type 1 (NF-1) [5]. These tumors are aggressive sarcomas of neuroectodermal differentiation and usually develop in deep soft tissue. Individuals with NF-1 have a significantly increased risk of developing MPNSTs frequently arising in preexisting neurofibromas which are uncommon in those without NF-1 [6]. Diagnosis may be delayed since this type of tumors is either asymptomatic or may cause minimal discomfort [7]. Furthermore, there are no standardized diagnostic criteria and the association with NF-1 is not always appreciated whereas no universally accepted management exists [6]. MPNSTs are extremely rare during childhood. In this report, we present 4 cases of MPNSTs in patients with NF-1 who were diagnosed in a single pediatric oncology unit during the last 15 years.

## 2. Material: Patients

Clinical, epidemiology, imaging findings as well as the management and outcome of our patients are shown in Table 1.

### 2.1. Patient 1

**Figure 1:**
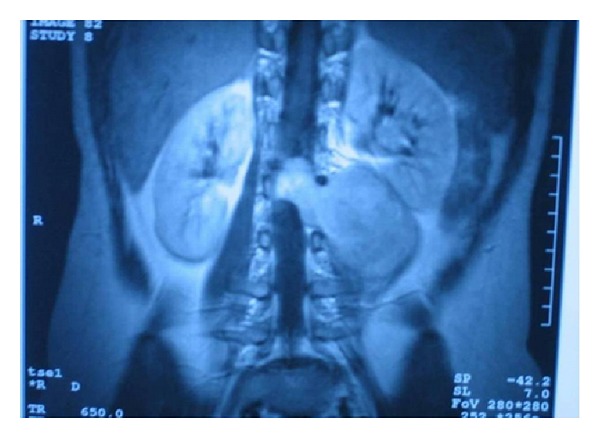
MRI revealed a huge mass adjacent to the first and second lumbar vertebrae with extension into the spinal canal.

A 13-year old girl with a known history of NF-1 was referred to our department with knee and back pain. She was short of stature whereas physical examination revealed café au lait spots in her body and scoliosis. Plain X-ray film revealed a lytic lesion on the second lumbar vertebra (body and arch) and magnetic resonance imaging (MRI) (Figure 1) revealed a huge mass adjacent to the first and second lumbar vertebrae with extension into the spinal canal. She was operated twice within a time period of two months for a decompression of spinal cord and a wide excision of the mass. A diagnosis of MPNST was established after histological investigation. Two months later, she presented with a local relapse and the mass was macroscopically removed during a third operation. Histological features were similar to the initial and adjuvant radiotherapy was applied. Seven months later she deteriorated with difficulty in walking, back pain, right coxalgia, left paresis, and abolition of knee and achilles tendon reflex as well as limited muscularis power and sensibility. MR images revealed local relapse and a palliative subtotal mass excision was undertaken. She finally died 32 months since diagnosis with progressive disease locally, neurological deterioration, paraplegia, frequent episodes of urinary tract infections, constipation, and ulcers decubiti.

### 2.2. Patient 2

**Figure 2:**
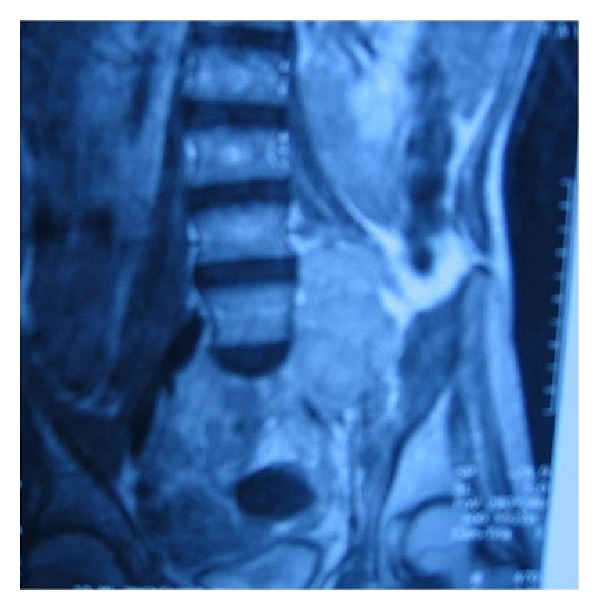
MRI revealed an intradural mass adjacent to the fifth lumbar vertebra, multiple pre- and paraspinal masses, and multiple masses of the left buttock.

A 13-year old girl with NF-1 was admitted to our department with an enlarging buttock mass and pain of the left leg (especially during the night), weakness in walking, and decreased sensation in toes which progressed to the whole left leg during a four-month period. Her history had started at the age of 10 years when she presented with a painless left buttock swelling. A biopsy was done and histology revealed a MPNST. She was operated and the buttock mass was macroscopically removed. Local radiotherapy was applied without any improvement. During the following months her disease progressed and she developed an intradural mass adjacent to the fifth lumbar vertebra, multiple pre- and paraspinal masses and multiple masses of the left buttock (Figure 2) as well as bilateral lung metastases. She received chemotherapy as per the SIOP MMT 98 protocol (4 cycles carboplatin/etoposide/vincristine and ifosfamide/etoposide/vincristine, alternatively) without any improvement. Palliative therapy was applied and she died 18 months since diagnosis with renal failure.

### 2.3. Patient 3

**Figure 3:**
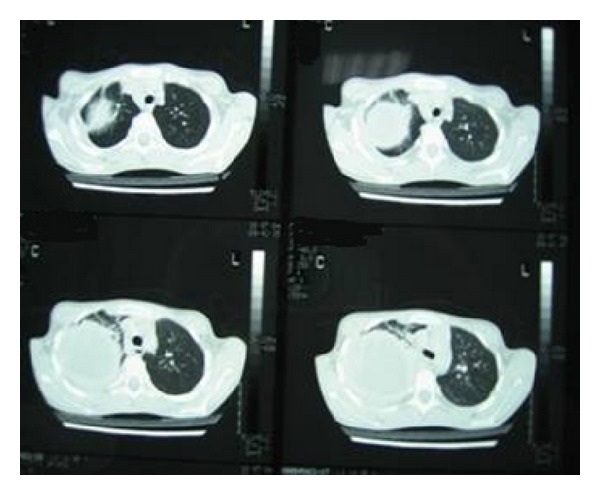
Computed tomography showed a large space-occupying solid and homogeneous right thoracic/mediastinal mass associated with pleural effusion.

A 9-year old boy with NF-1 was referred to our department with low grade fever and right thoracic pain. Initial imaging studies showed a large space-occupying solid and homogeneous right thoracic/mediastinal mass associated with pleural effusion (Figure 3). He was operated and a huge mass was macroscopically removed. Histology showed MPNST. The child was treated with local radiotherapy but tumor started growing locally while receiving RT which, therefore, was not completed. He then received 3 courses of chemotherapy (IVA: vincristine, actinomycin D, and ifosfamide) as per the SIOP MMT 98 protocol without any improvement. On the contrary, there was visible and palpable anterior chest wall mass which was considered inoperable. Our patient received palliative therapy and died 10 months since diagnosis.

### 2.4. Patient 4

**Figure 4:**
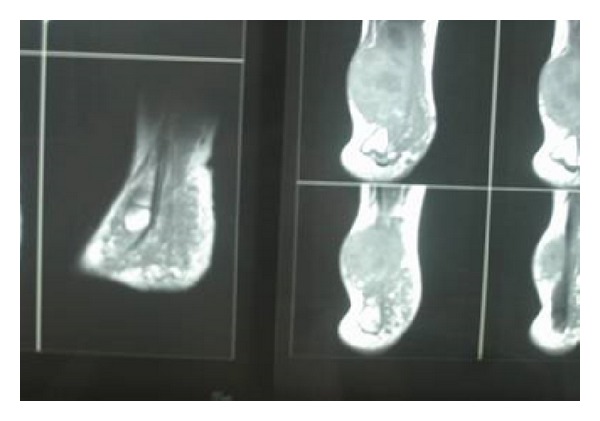
MR images showed the presence of a huge mass of the right calf and foot.

A 12-year old girl with NF-1 was admitted to our department with right tibia and tibiotarsal swelling, severe pain, and difficulty in walking. She was first examined by her paediatrician when she was 10 years old and a painless enlarging mass in her right foot was found. MR images showed the presence of a huge mass of the right calf and foot (Figure 4). A subtotal excision was performed and histology showed MPNST. Local radiotherapy and physical therapy were applied after the operation. During follow-up, 5 months later, foot disease was stable but lung metastases had developed. Fine needle biopsy and cytology of pleural fluid revealed disease progression. She then received 7 cycles of chemotherapy (doxorubicin/ifosfamide and vincristine/etoposide/ifosfamide, alternatively) without any improvement. Palliative therapy was used and she died 22 months since initial diagnosis.

## 3. Discussion

Individuals affected by NF-1 have an increased risk of developing both benign and malignant tumors [6]. NF-1 is an autosomal dominant neurocutaneous inherited genetic disorder, with an estimated birth incidence of 1 in 2500 to 3000 live births [8, 9]. The diagnosis of NF-1 is made if two or more of the following signs are present: café au lait spots with a diameter of 5 mm or greater (before puberty) or 15 mm or greater (after puberty), two or more neurofibromas of any type or one plexiform neurofibroma, skinfold freckling, optic glioma, two or more Lisch nodules, characteristic osseous dysplasia, and first degree relatives with NF-1 [8, 10].

NF-1 is associated with alterations in the NF-1 gene which is a tumor suppressor gene located in the proximal long arm of chromosome 17 and encodes a protein called neurofibromin [11]. Patients with a microdeletion of the NF-1 locus have higher numbers of discrete dermal neurofibromas at earlier ages and might have a higher incidence of MPNSTs than the overall NF-1 population [12]. It is not clear whether the genetic alterations reported in MPNSTs are causally related to tumor genesis or malignant transformation. Moreover, it is not known whether a specific form of therapy is likely to be more effective in a subset of patients with these changes [6]. Nevertheless, since p53 reactivity is obtained in more than half of MPNSTs but not in neurofibromas, the role of the functional loss of p53 gene in molecular pathogenesis of MPNSTs is remarkable [8, 13]. Unfortunately, no genomic data is available concerning our patients. In the future, the routine evaluation of tumor suppressor genes or oncogenes might be a standard practice in the management of MPNSTs [6].

The most common tumor in individuals with NF-1 is the neurofibroma, a heterogeneous peripheral nerve sheath tumor [14]. Although neurofibromas are benign, a minority of patients with NF-1 show an increased incidence of malignancy such as MPNSTs, astrocytomas, and leukemias [8, 11, 15, 16].

Most NF-1-associated MPNSTs appear to arise within preexisting plexiform neurofibromas. Patients with NF-1 are at greatest risk for developing sarcomas 10–20 years after the appearance of neurofibromas and, therefore, MPNSTs are frequently detected in adults 20–50 years of age. This type of tumors accounts for approximately 5–10% of all soft tissue sarcomas during adulthood whereas in more than 50% of these patients NF-1 also coexists [1, 2, 4, 6, 8, 15, 17–19]. It is of note that the incidence of MPNSTs in patients with NF-1 is estimated to be 2–5% compared to 0.001% in the general population [1] and, consequently, individuals with NF-1 should be followed closely [2]. MPNSTs arising in people with NF-1 are usually diagnosed at an earlier age than in the general population and have been reported to carry a worse prognosis than those arising in patients without NF-1 [1, 2, 9, 20]. A male predominance is reported in some studies [1, 15, 20] whereas other authors found that females seem to be involved equally to males [21]. Among our patients, all were affected by NF-1 and had a family history of NF-1 whereas three of them were girls and one boy.

All of our patients presented with pain and an enlarging mass. In the NF-1 patients with MPNSTs, an enlarging mass and pain are the initial reason for asking for medical advice [1, 4, 20, 21]. The most commonly affected sites include the proximal parts of the upper and lower extremities [3, 4, 22, 23]. However, some authors have reported the head and neck or the trunk as common sites [1, 3, 8, 24]. On the other hand, many studies reported that foot and lung are a rare localization [1, 3, 4]. In our patients, MPNSTs were located in the buttock, the spinal cord, the lung, and the foot (heel).

MPNSTs are often difficult to detect because the clinical characteristics of malignancy may be similar to active, benign plexiform neurofibromas [6]. Forty four percent of plexiform neurofibromas are diagnosed before the age of five years. They develop along a nerve and may involve multiple branches, nerve roots, and plexi. These tumors may infiltrate the surrounding structures causing soft tissue and bone hypertrophy and, consequently, functional compromise [25]. Clinicians should be alerted to the presence of MPNSTs when a patient with NF-1 develops unremitting pain not otherwise explained, rapid increase in size of a plexiform neurofibroma, change in consistency from soft to hard, or a neurological deficit [6].

Concerning imaging investigations, our patients were evaluated with MRI (three of them) and CT (the fourth one). Imaging investigations are important in order to evaluate the site, extent, and change in size of a neurofibroma but are not reliable to detect a malignant transformation [6]. Positron emission tomography (18-FDG PET) has been proposed as a potentially useful, noninvasive method for detecting that malignant change in patients with NF-1 [26]. However, the use of this technique in order to detect the malignant transformation is not always effective in these at-risk patients [6].

Regardless of the imaging findings, a biopsy was necessary for the diagnosis in our patients. For the histological diagnosis Tru-Cut needle biopsy is reported as the method of choice [6]. A technique of a targeted biopsy using 18-FDG PET is being developed and may be of potential benefit [6]. Histologically, MPNSTs are characterized by mild to significant hypercellularity, nuclear atypia, and increased mitotic index [1]. Other pathological criteria for malignancy include invasion of surrounding tissues by tumor cells, vascular invasion, marked nuclear pleomorphism, and necrosis [27]. Actually, there is a histological spectrum ranging from clearly benign to clearly malignant [6, 27–29].

Most MPNSTs are aggressive, high grade sarcomas with a high likelihood of local recurrence, and distant metastases [1, 24]. The local recurrence rate varies from 40% to 65% and the metastatic rate from 40% to 68% [8]. They frequently metastasize to the lungs followed by bone whereas lymph node metastases are uncommon [4, 8, 24]. Other sites of metastasis include liver, brain, soft tissue, skin, and retroperitoneum [1]. Two of our patients developed lung metastases.

MPNSTs have poor outcome if untreated [1]. These tumors are relatively resistant to chemotherapy and radiation therapy and, therefore, complete surgical excision continues to be the gold standard for treatment [2, 3, 24, 30]. Chemotherapy is usually preferred for metastatic disease. Doxorubicin or a combination of doxorubicin and ifosfamide has been shown to be effective but long-lasting remissions are not frequent. Chemotherapy may be also useful in the preoperative management in order to decrease the size in patients with inoperable tumors [30]. On the other hand, supplemental radiation therapy is recommended for positive microscopic margins providing local control and delaying the onset of recurrence [6, 24].

MPNSTs have a relatively poor prognosis in comparison with other soft-tissue malignancies. The five-year survival for adults and children varies from 34% to 44% [1–3, 18, 22, 24, 31–33]. All of our patients died of disease progression in spite of a wide excision and additional chemotherapy and radiotherapy. A difference concerning the outcome has been detected between patients with and without NF-1. Patients with MPNST in the absence of NF-1 were reported to have a five-year survival of 50% whereas in the presence of NF-1 the survival rates are reduced with a five-year survival of 15% or 9% in case of large tumors [1]. On the contrary, other studies showed no difference between patients with or without NF-1 [2].

Regarding the prognostic factors, although patients with tumors in the extremities had a better prognosis than those in head and neck, location did not appear to be a clear prognostic factor [1, 20, 21, 23]. The most significant prognostic factor appears to be the gross tumor resectability which increased the five-year survival to 65% [3]. Patients who had local excisions, wide local excisions, or amputations had longer disease-free survival rates than those who had subtotal resections and, consequently, the aim of surgery should be the complete removal of the lesion with tumor-free margins. The prognostic pathology parameters include also tissue necrosis, high cellularity, and increased mitotic index. Other factors associated with decreased survival rates include a size of over 5 cm and tumor recurrence [1–4, 22, 33–36].

In conclusion, MPNSTs arising in patients with NF-1 are high grade sarcomas with dismal prognosis. The individuals with NF-1 should be followed closely in order to identify timely the malignant transformation. Despite the malignant potential, these tumors may originally present as a benign-appearing mass [37]. MPNSTs should be managed by a team of specialists in neurology, genetics, surgery, and oncology. Aggressive surgery and complete, wide excision improve disease-free survival rates whereas the role of chemotherapy and radiotherapy is not clear although all patients will receive radiation therapy at some stage of the disease. In this report we presented 4 cases of MPNSTs. Wide excision of the tumor and radiotherapy were applied to all and adjuvant chemotherapy was given to three of them after disease had progressed. All four died with progressive disease locally and pulmonary metastases in two of them.

## Figures and Tables

**Table 1:** Characteristics, treatment, and outcome of patients.

Case	Age∗	Gender	Symptoms/signs	NF-1	Location	Size∗∗	Treatment	Outcome
1st	13	Female	Pain, mass	Yes	Spinal cord	8 × 7 × 5 (MRI)^1^	Wide excision of the tumor and RT	Death
2nd	13	Female	Pain, mass	Yes	Buttock	6 × 4 × 6 (MRI)^2^	Wide excision of the tumor and RT-CT	Death
3rd	9	Male	Pain, mass	Yes	Trunk	Huge (CT scan)^3^	Wide excision of the tumor and RT-CT	Death
4th	12	Female	Pain, mass	Yes	Foot	10 × 3.5 × 5.5 (MRI)^4^	Wide excision of the tumor and RT-CT	Death

*years, **cm, 1: Figure 1, 2: Figure 2, 3: Figure 3, 4: Figure 4, RT: radiotherapy, CT: chemotherapy.
